# Real-Time Forecasting of the COVID-19 Outbreak in Chinese Provinces: Machine Learning Approach Using Novel Digital Data and Estimates From Mechanistic Models

**DOI:** 10.2196/20285

**Published:** 2020-08-17

**Authors:** Dianbo Liu, Leonardo Clemente, Canelle Poirier, Xiyu Ding, Matteo Chinazzi, Jessica Davis, Alessandro Vespignani, Mauricio Santillana

**Affiliations:** 1 Computational Health Informatics Program Boston Children’s Hospital Boston, MA United States; 2 Department of Pediatrics Harvard Medical School Boston, MA United States; 3 Tecnologico de Monterrey Monterrey Mexico; 4 Harvard TH Chan School of Public Health Boston, MA United States; 5 Laboratory for the Modeling of Biological and Socio-technical Systems Northeastern University Boston, MA United States; 6 ISI Foundation Turin Italy

**Keywords:** COVID-19, coronavirus, digital epidemiology, modeling, modeling disease outbreaks, emerging outbreak, machine learning, precision public health, machine learning in public health, forecasting, digital data, mechanistic model, hybrid simulation, hybrid model, simulation

## Abstract

**Background:**

The inherent difficulty of identifying and monitoring emerging outbreaks caused by novel pathogens can lead to their rapid spread; and if left unchecked, they may become major public health threats to the planet. The ongoing coronavirus disease (COVID-19) outbreak, which has infected over 2,300,000 individuals and caused over 150,000 deaths, is an example of one of these catastrophic events.

**Objective:**

We present a timely and novel methodology that combines disease estimates from mechanistic models and digital traces, via interpretable machine learning methodologies, to reliably forecast COVID-19 activity in Chinese provinces in real time.

**Methods:**

Our method uses the following as inputs: (a) official health reports, (b) COVID-19–related internet search activity, (c) news media activity, and (d) daily forecasts of COVID-19 activity from a metapopulation mechanistic model. Our machine learning methodology uses a clustering technique that enables the exploitation of geospatial synchronicities of COVID-19 activity across Chinese provinces and a data augmentation technique to deal with the small number of historical disease observations characteristic of emerging outbreaks.

**Results:**

Our model is able to produce stable and accurate forecasts 2 days ahead of the current time and outperforms a collection of baseline models in 27 out of 32 Chinese provinces.

**Conclusions:**

Our methodology could be easily extended to other geographies currently affected by COVID-19 to aid decision makers with monitoring and possibly prevention.

## Introduction

First detected in Wuhan, China, in December 2019, severe acute respiratory syndrome coronavirus 2 (SARS-CoV-2) infection had rapidly spread by late January 2020 to all Chinese provinces and many other countries [1-4]. On January 30, 2020, the World Health Organization (WHO) issued a Public Health Emergency of International Concern (PHEIC) [5-8]; and on March 11th, the WHO declared the coronavirus disease (COVID-19) a pandemic [5]. By April 18, 2020, the virus had affected more than 2,300,000 people and caused the deaths of 150,000 in more than 180 countries [7].

In the last decade, methods that leverage data from internet-based data sources and data from traditional surveillance systems have emerged as a complementary alternative to provide near real-time disease activity estimates (eg, for influenza and dengue) [9-13]. Despite the fact that these methodologies have successfully addressed delays in the availability of health reports as well as case count data quality issues, developing predictive models for an emerging disease outbreak such as COVID-19 is an even more challenging task [14]. There are multiple reasons for this; for example, the availability of epidemiological information for this disease is scarce (there is no historical precedent about the behavior of the disease); the daily/weekly epidemiological reports that become available are frequently revised and corrected retrospectively to account for mistakes in data collection and reporting (a common practice in public health reports); and the presence of a diverse array of uncertainties about disease burden due in part to underreporting of cases [15].

Most efforts to estimate the time evolution of COVID-19 spread and the effect of public health interventions have relied on mechanistic models that parameterize transmission and epidemiological characteristics to produce forecasts of disease activity [16,17]. In contrast, only a limited number of studies have investigated ways to track COVID-19 activity, leveraging internet search data [1,13,18], and few to the best of our knowledge have combined internet-based data sources and mechanistic estimates to forecast COVID-19 activity [19].

We present a novel hybrid methodology that combines mechanistic and machine learning methodologies to successfully forecast COVID-19 in real time at the province level in China [20,21]. We used a data-driven approach to incorporate inputs from (a) official health reports from Chinese Center for Disease Control and Prevention (China CDC), (b) COVID-19–related internet search activity from Baidu, (c) news media activity reported by Media Cloud, and (d) daily forecasts of COVID-19 activity from the simulation epidemiological model GLEAM (global epidemic and mobility), a metapopulation mechanistic model [16]. Inspired by a methodology previously used to successfully forecast seasonal influenza in the United States at the state level [11] and previous methods to monitor emerging outbreaks [22,23], our method is capable of reliably forecasting COVID-19 activity even when limited historical disease activity observations are available. From a methodological perspective, the novelty in our approach comes from a clustering technique that enables the exploitation of geospatial synchronicities of COVID-19 activity across Chinese provinces and a data augmentation technique to mitigate the scarcity of historical data for model training.

## Methods

### Experimental Design

Our method was designed for forecasting COVID-19 2 days ahead of the current time. We used as inputs the following data sources: COVID-19 activity reports from China CDC; internet search frequencies from Baidu; a number of related news reports from 311 media sources, as reported by the Media Cloud platform; and COVID-19 daily forecasts from a metapopulation mechanistic model. Our machine learning methodology also used a clustering and data augmentation technique. We provide details about data sources and statistical methods in the following sections.

### Data Sources

#### Daily Reports of COVID-19

Case counts of COVID-19 were obtained from China CDC. These data are curated and publicly available via the Models of Infectious Disease Agent Study (MIDAS) association [24]. All data were collected on the original date they became available. Indeed, case counts released by China CDC can be revised, up to several weeks later. In this study, we only used unrevised data, which is the real case scenario to produce real-time estimates. The reports, available for all the provinces, include various activity trends such as new diagnosed cases, new suspected cases, and new reported deaths. For our study, we selected the number of confirmed cases as the epidemiological target and collected activity reports from January 10, 2020, to February 21, 2020.

#### Baidu Internet Search Activity: Data Exclusion

**Figure 1:**
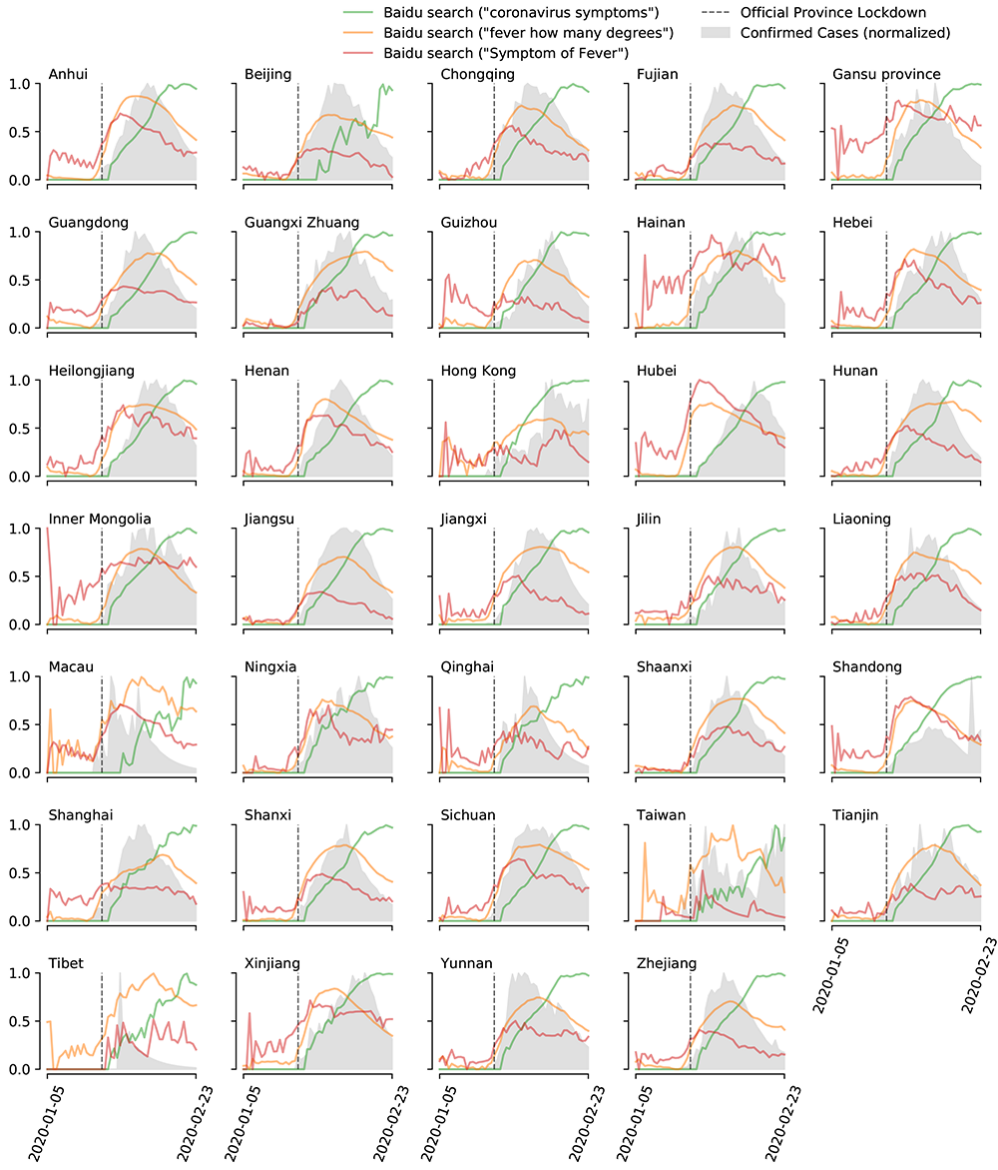
Visualization of the evolution of coronavirus disease (COVID-19) cases and Baidu search trends. The evolution of COVID-19 cases is represented in gray and Baidu search trends in green and orange. All-time series have been smoothed for visualization purposes.

We collected the daily search fraction for three different COVID-19–related search terms in Mandarin (“COVID-19 symptoms” [“新冠肺炎症状”], “how many degree is fever” [“多少度算发烧”], and “symptoms of fever” [“发烧症状”]). These terms were selected based on their correlation and potential association with case counts of COVID-19 [25] and collected individually for each province from January 1, 2020, to February 21, 2020. Our decision to use internet activity as a source of information is based on the hypothesis that search frequencies from COVID-19–related keywords reflect, to an extent, the number of people presenting symptoms related to COVID-19 before their arrival at a clinic. Given Baidu imposes limits to data access for researchers, we were unable to conduct a broad analysis on a wide range of keywords. A visualization of the Baidu search term time series can be seen in Figure 1.

#### News Reports

An online open-source platform called Media Cloud, which allows the tracking and analysis of media for any topic of interest through the matching of keywords, was used. We obtained volumes of the number of news articles available over time from a collection of 311 Chinese media websites using the keywords “coronavirus,” “COVID-19,” “2019-nCoV,” “pneumonia,” “fever,” “cough,” and the name of each province to generate province-specific news activity trends. Media data from January 1, 2020, to February 21, 2020 were collected and used as additional source information.

#### Global Epidemic and Mobility Model

The global epidemic and mobility model, GLEAM, is an individual-based, stochastic, and spatial epidemic model [26-28] that has been used to simulate the early stages of the COVID-19 epidemic in mainland China and across the world [16]. GLEAM is based on a metapopulation approach in which the world population is divided into subpopulations centered around major transportation hubs (usually airports). Over 3000 subpopulations in about 200 different countries and territories are included in the model. The subpopulations are connected by short-range commuting and long-range travel networks that determine the flow of individuals traveling daily among them. Short-range mobility patterns (eg, daily commuting) are derived from data collected from the National Statistical Offices of 30 countries on five continents [26]. In addition, for the COVID-19 epidemic, mobility variations in mainland China are further calibrated using deidentified and aggregated domestic population movement data as derived from Baidu Location-Based Services. The airline transportation data consider daily origin-destination traffic flows obtained from the Official Aviation Guide and the International Air Transport Association databases (updated in 2019), and accounting for travel restrictions in 2020. Within each subpopulation, the human-to-human transmission of COVID-19 is modeled using a compartmental representation of the disease where each individual can occupy one of the following four states: susceptible (S), latent (L), infectious (I), and removed (R). Susceptible individuals can acquire the virus through contacts with individuals in the infectious state, and become latent, meaning they are infected but cannot transmit the infection yet. Latent individuals progress to the infectious stage with a rate inversely proportional to the latent period. Infectious individuals progress into the removed stage with a rate inversely proportional to the infectious period. Removed individuals represent those who can no longer infect others, meaning they were isolated, hospitalized, died, or have recovered.

The model produces an ensemble of possible epidemic scenarios providing epidemic indicators, such as the number of newly generated infections and deaths in each subpopulation. The model is initialized by a starting date of the epidemic between November 15, 2019, and December 1, 2019, with 20 to 40 cases caused by zoonotic exposure [29-32]. The transmission dynamic is calibrated by using an Approximate Bayesian Computation approach to estimate the posterior distribution of the basic reproductive number R_0_ that uses as evidence the detection of infections imported from China at international locations across the world [33-37]. A sensitivity analysis has been performed on the initial conditions of the model considering different values for the mean latency period (range 3-6 days), the mean infectious period (range 2-8 days), the generation time (range 6-11 days), and the initial number of zoonotic cases (range 20-80). The calibrated model is then used to generate the out-of-sample ensemble of stochastic epidemic evolutions across mainland China.

### Statistical Analysis

#### Aggregation of Daily Reports

To enhance signal and reduce noise, we aggregated case count, search volumes, and media article count for each *δt* = 2 days window.

As COVID-19 is an emerging outbreak, the amount of epidemiological information, either official or unofficial, is low, and thus, limits our capacity to build predictive models. To maximize usage of data, we applied the strategies below.

#### Clustering

We clustered the 32 provinces into several groups and trained a model for each group. Clustering and model retraining processes were repeated on every single new prediction date. To determine the similarities in outbreak patterns across Chinese provinces, we calculated the pairwise correlation matrix for confirmed COVID-19 cases by using all historical data available. Then, based on similarity matrix, provinces were clustered by using complete linkage hierarchical clustering, which is an agglomerative hierarchical clustering method, creating clusters based on most dissimilar pairs [38]. The number of clusters K was determined by choosing the K, thereby maximizing the Calinski-Harabasz index [39]. Our clustering method gained higher stability when more data points were available for clustering [40]. More details of the clustering method are presented in Multimedia Appendix 1 [41-43].

#### Data Augmentation

We conducted data augmentation by using a bootstrap method to resample each data point of the training data set. We made 100 bootstrap samples for each data point to which we added a random Gaussian noise with a mean of 0 (SD 0.01). Due to the stochasticity of both the clustering algorithm and the model training processing, on each prediction day, we run the whole clustering-training process 20 times and take an average of the outputs as our final prediction. Our multistep approach may introduce stochasticity in three different steps: (a) the clustering process, (b) the data augmentation process, and (c) the regression algorithm. To ensure robustness of our prediction results, the whole process (from clustering to out-of-sample prediction) on each prediction date was repeated at least 20 times and the ensemble (via an averaging approach) predictions were reported as the final prediction. We chose to use an empirical approach to explore whether the number of computational experiments were sufficient to lead to a stable performance. In order to achieve this, we conducted ensemble prediction experiments using realizations from 1 to 50 prediction efforts. We documented the performance of these ensemble predictions using root mean square error (RMSE) and correlation in (Multimedia Appendix 2, Table S1). The performance of the ARGONet + GLEAM method plateaued after about 10-15 realizations as seen on this table. Therefore, we concluded that 20 realizations of our algorithm was an adequate number to ensure robustness and stability of the prediction while not imposing too much computational burden.

#### Predictive Model

For our prediction task, we fitted a LASSO (least absolute shrinkage and selection operator) multivariable regularized linear model for every data set generated from our clustering and augmentation steps at time t.

The LASSO technique minimizes the mean squared error between observations and predictions subject to a L1 norm constraint (more details of this method are provided in Multimedia Appendix 1). The number of new confirmed COVID-19 cases for the next bi-day can be then expressed as:


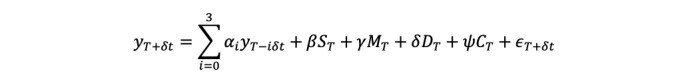


where *y_T + δt_* is the estimate at date *T + δt*; *δt* = 2 days; *y_T_* is the number of cases at date *T*; *S_T_* is the search volume at date *T; M_T_* is the number of media articles at date *T*; *D_T_* is the number of deaths at date *T*; *C_T_* is the number of cumulative cases at date *T*; and *ϵ_T + δt_* is the normally distributed error term.

Models were dynamically recalibrated, similar to the method presented by Santillana et al [44] and Lu et al [11]. Our method, ARGONet + GLEAM, was implemented in an R 3.5.3 environment with a glmnet 3.0-2 library.

**Figure 2:**
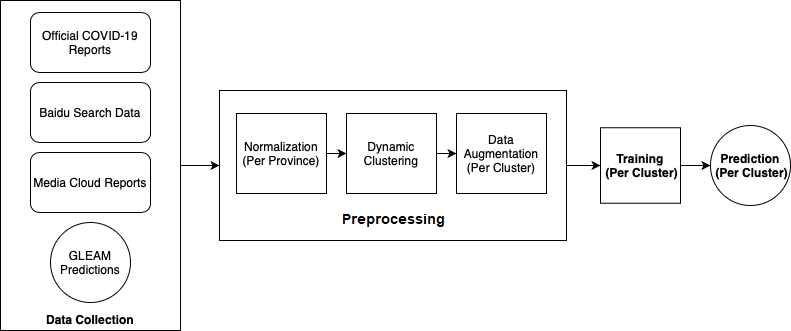
Summary of the methods used to obtain our coronavirus disease (COVID-19) estimates. GLEAM: global epidemic and mobility.

A summary of our method can be seen in Figure 2.

#### Performance of Model and Relevance of Predictors

Two different metrics were used to measure the performance of ARGONet + GLEAM: (1) the RMSE and (2) the Pearson correlation. To assess the predictive power of our methodology, we compared our performance against the following models:

1. Persistence rule (baseline): a rule-based model that uses the new case count at date *T* as an estimate of the prediction for *T*+*δt* so that *yT*+*δt = yT*
2. Autoregressive (AR): a simple AR model built on COVID-19 cases that occurred in the previous three AR lags (2-day reports) (see Multimedia Appendix 1 for more information on this model)
3. ARGONet: an alternate version of our methodology that does not include any mechanistic information but including clustering and data augmentation approaches.

As linear models are used in this study, the relevance of predictors in predicting new cases can be defined thanks to the associated factor of each term in the trained model. As all data were normalized using the z-score (strictly within the training data sets) during training and prediction, the associated factor can be approximately understood as how many standard deviations the predicted new cases *y_T_ + δt* will change if 1 standard deviation changes in the predictor.

### Data Sharing

All codes and data will be made available via the Harvard dataverse.

## Results

**Figure 3:**
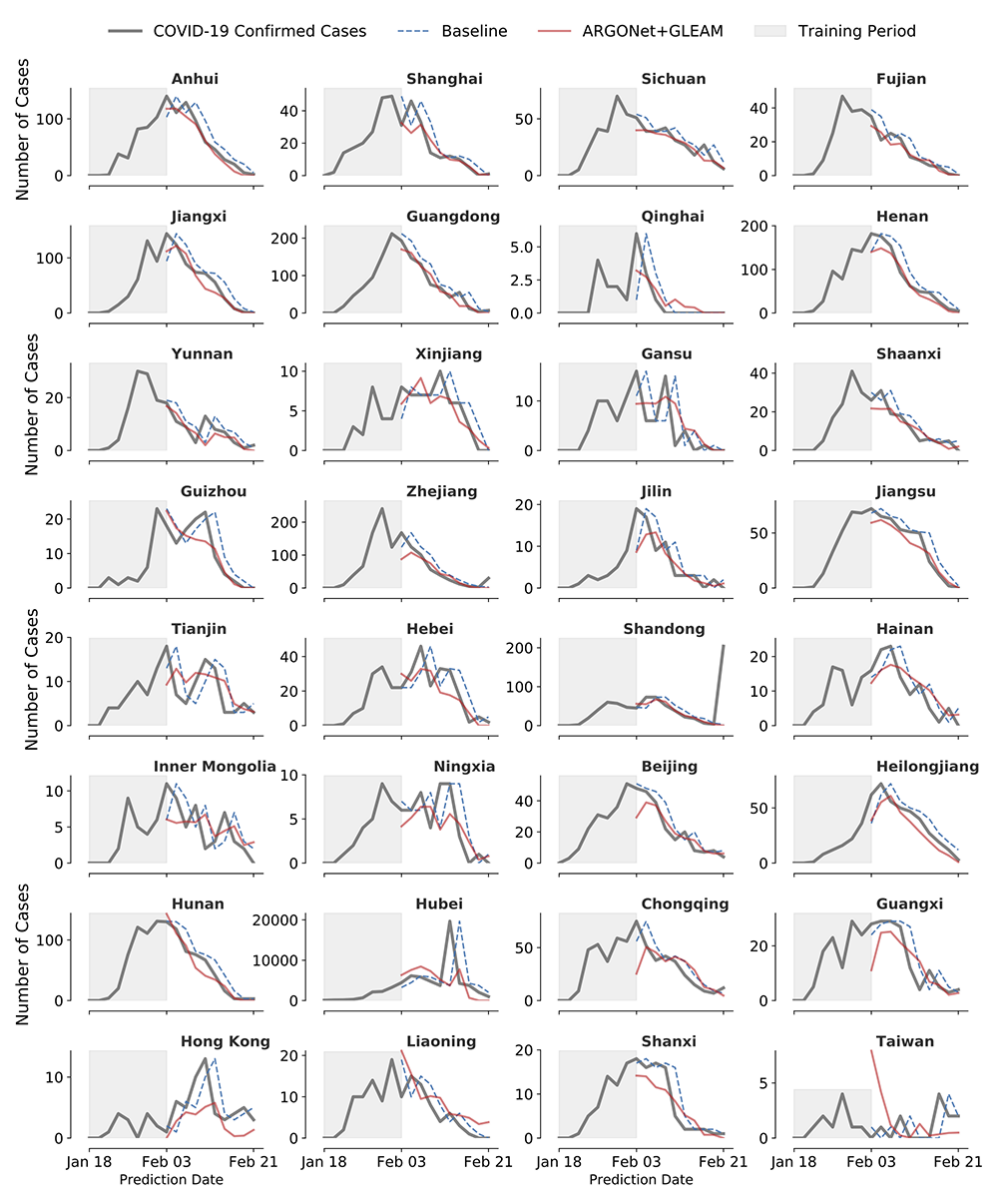
Graphical visualization of the estimates obtained by ARGONet + GLEAM. The number of new confirmed cases for coronavirus disease (COVID-19), as reported by China CDC (solid black), along with ARGONet + GLEAM (solid red) 2-day, ahead-of-time estimates between February 3, 2020, to February 21, 2020. As a comparison, the dotted blue line represents the persistence model.

We produced 2-day-ahead (strictly out-of-sample) and real-time COVID-19 forecasts for 32 Chinese provinces for the time period spanning February 3, 2020, to February 21, 2020. A visual representation of our out-of-sample model forecasts is shown in Figure 3 along with the subsequently observed COVID-19 cases, as reported by China CDC.

Our results show that ARGONet + GLEAM outperforms the persistence model in 27 out of 32 Chinese provinces. Even in provinces where ARGONet + GLEAM failed to produce improvements to the baseline model, our model produced reasonable disease estimates as seen in Figure 3. These provinces include Shanxi, Liaoning, Taiwan, Hong Kong, and Guangxi (the latter three with very different administration, and likely health care, systems compared to the rest of the provinces).

### Experimental Design AR Model

**Figure 4:**
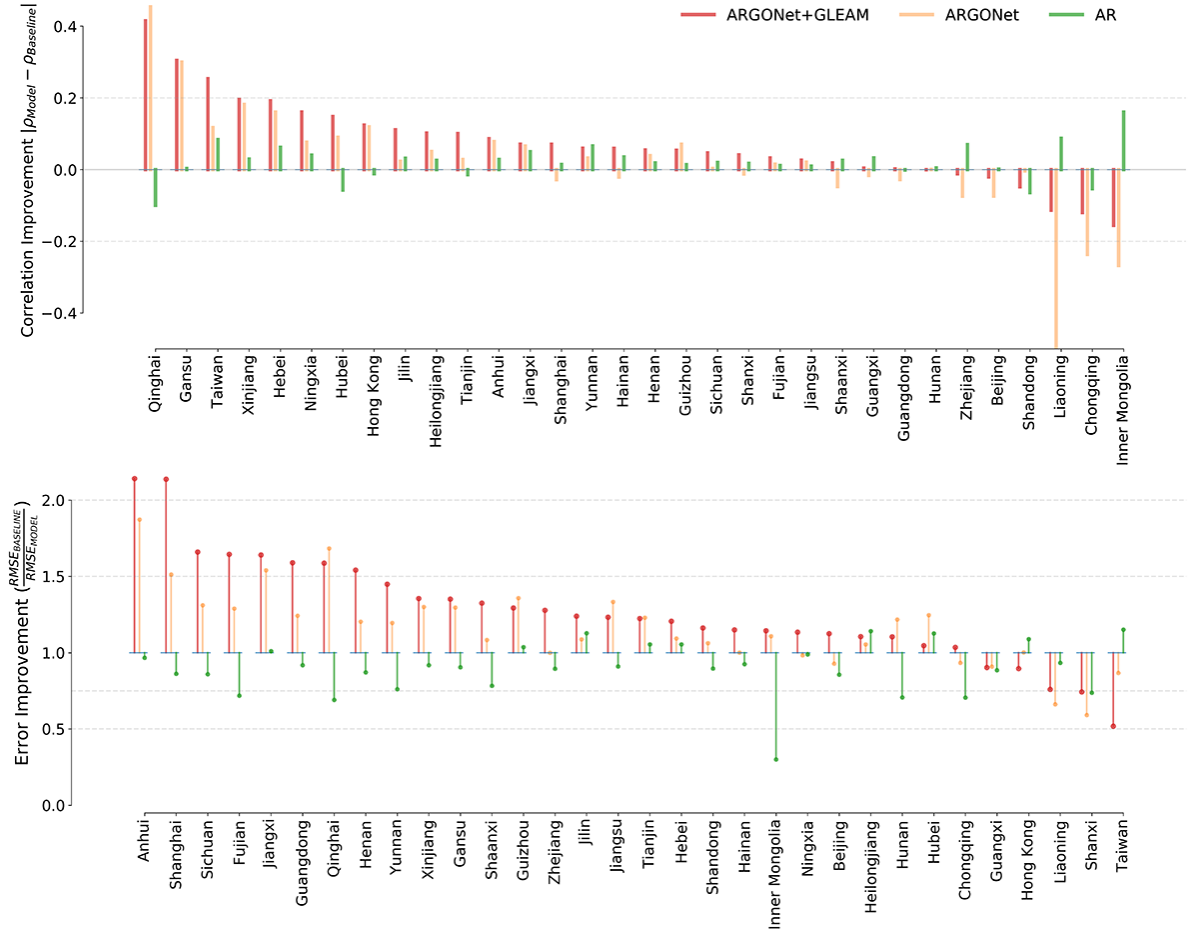
Graphical visualization of the models’ performances. Comparison of the improvement in terms of root mean square error (RMSE) (top) and Pearson correlation (bottom) for each model used in the study. To facilitate comparison between model scores in each province in terms of RMSE, we normalized the RMSE score of each model by the baseline’s RMSE and visualized its inverse value. In this way, scores above one imply an improvement (RMSE reduction), whereas a score below one implies the model had a bigger RMSE in comparison to the baseline. In the case of correlation, we plotted the difference between the absolute values between each model’s correlation and the baseline. Each panel is ordered, from left to right, based on the metric performance of ARGONet + GLEAM (solid red). AR: autoregressive.

We analyzed the performance of models built using only local, province-level epidemiological data as input. We generated an AR model for each province, built on COVID-19 cases that occurred in the previous three AR lags (ie, the previous three 2-day reports), and compared our estimates with the baseline. Our results, presented in Figure 4 (also see Tables S2 and S3 in Multimedia Appendix 2 for a detailed description of our model results), labeled AR, show that the AR model’s predictive power was overall inferior to baseline performance, with exception to Jilin, Tianjin, Hebei, Hubei, and Heilongjiang. Subsequently, we incorporated local disease-related internet search information from Baidu and news alert data from Media Cloud as inputs to build ARGO-type models [9]. These ARGO-type models showed marginal predictive power improvements when compared with AR models and only outperformed the baseline in seven provinces.

### Dynamic Clustering of Chinese Provinces

**Figure 5:**
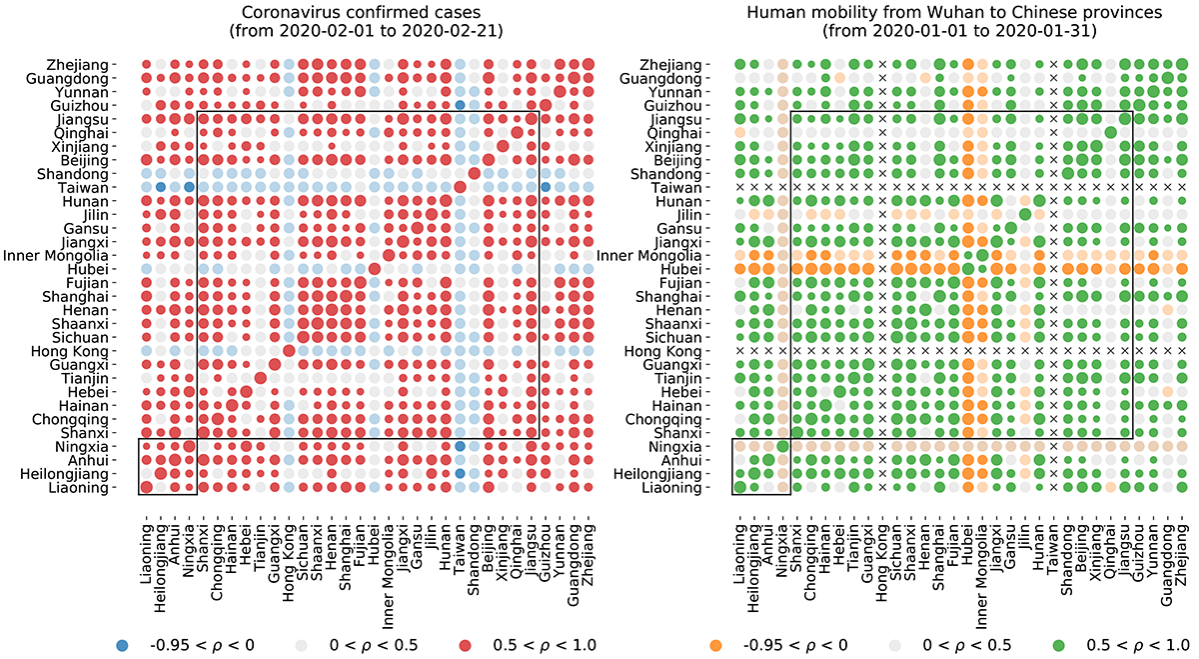
Visualization of the pairwise correlation matrices of confirmed cases and human mobility from Wuhan to each Chinese province. During the period of January, we can see a similar trend of mobility for a big cluster of provinces as well as a similar trend of number of confirmed cases for the period of February.

Based on prior work on influenza activity prediction [11], we added historical COVID-19 activity information for all Chinese provinces to the input of our local models. We calculated the pairwise correlation matrix for confirmed COVID-19 cases between all Chinese provinces, between February 1 and February 21, 2020 (Figure 5). Our results showed that most of the provinces experienced similar epidemic trends. To build our (clustered) predictive models, we combined the data available from several provinces with similar trends (in terms of correlation, which was strictly calculated within our training period at the time-step of prediction). The clustering modeling approach, which incorporated internet-based data sources as the ARGO-type models, produced forecasts that led to error reductions for 17 out of 32 provinces compared to the persistence model and improved correlation values in 20 out of 32 provinces.

### Data Augmentation

As an additional way to increase the number of observations in the training set of each cluster, we implemented a data augmentation technique. This process consisted of generating new observations via a Bootstrap method and addition of random Gaussian noise 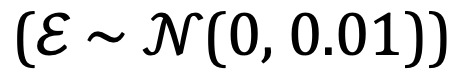 to every randomly selected observation.

### ARGONet Model

**Figure 6:**
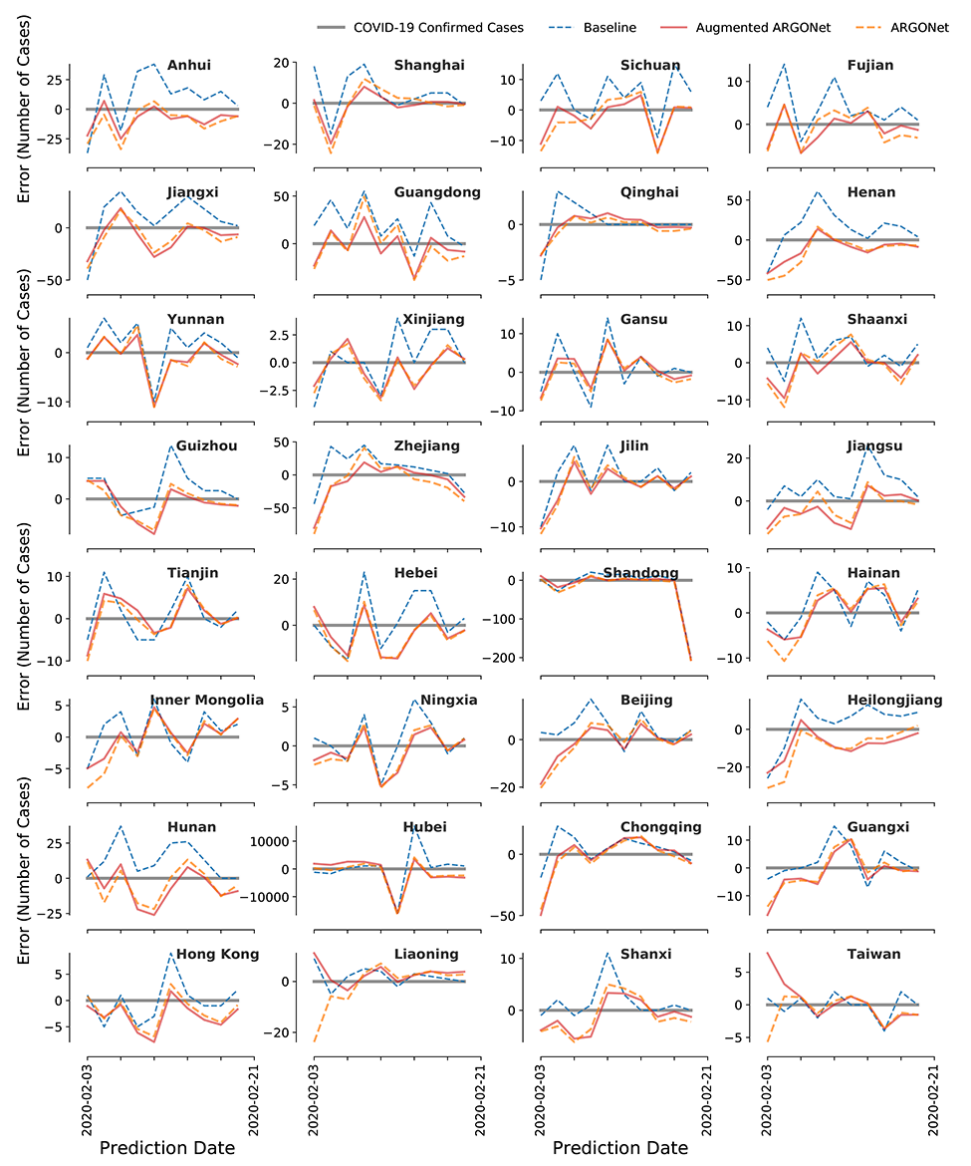
Visualization of the errors. Graphical visualization of the out-of-sample coronavirus disease (COVID-19) error (ŷ–y) between February 3, 2020, and February 21, 2020.

The results of incorporating both clustering and augmentation techniques can be seen in Figure 4 and a visualization of the errors can be seen in Figure 6. For simplicity, we labeled these predictions ARGONet, even though this implementation of ARGONet is an enhanced version specifically designed for emerging outbreaks where data are scarce. In terms of RMSE, our results show that ARGONet’s predictive power was able to outperform AR and the persistence model in 25 of the 32 Chinese provinces. In terms of correlation, ARGONet outperformed the baseline (persistence) model in 18 provinces.

### ARGONet + GLEAM Model

We included forecasts produced by mechanistic model as an additional input in our models (prior to the clustering and augmentation steps). The results of incorporating these estimates can be seen in Figure 4 with the name of ARGONet + GLEAM and a visualization of the errors can be seen in Figure 6. Our results show that the inclusion of mechanistic model estimates improved ARGONet’s predictive power across most provinces. ARGONet + GLEAM led to error reductions in 27 out of 32 provinces compared to the baseline. In terms of correlation, it improved in 26 out of 32 provinces. Provinces like Qinghai, Hunan, and Jiangxi showed the biggest improvement, whereas Taiwan, Hong Kong, Shanxi, and Liaoning did not display error reductions.

### Visualization of the Results

**Figure 7:**
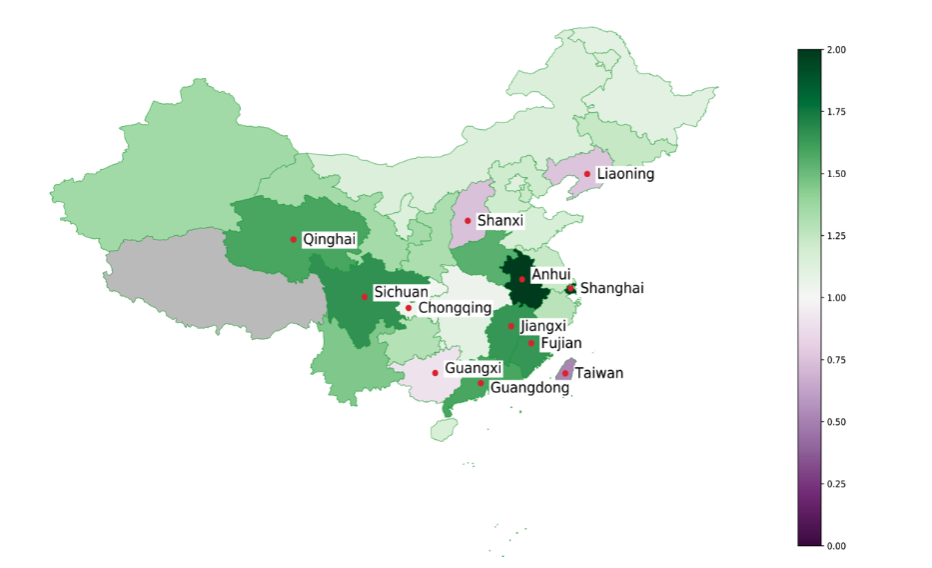
Geographical visualization of the relative improvement of ARGONet + GLEAM compared to the baseline. Chinese provinces that show an increase in performance relative to the baseline are shaded green, while provinces that did not perform better than our baseline are shaded purple. Provinces with the highest improvement (Anhui, Shanghai, Sichuan, Fujian, Jiangxi, Guangdong, and Qinghai) and underperformance (Taiwan, Shanxi, Liaoning, Hong Kong, and Guangxi) are identified by a red dot over the province.

As an alternative way to visualize ARGONet + GLEAM’s predictive performance, we plotted a map with Chinese provinces (Figure 7), color coded based on the improvement shown in Figure 4. From a geographical perspective, the provinces where ARGONet + GLEAM had the most improvement (Anhui, Jiangxi, Fujian, Sichuan, and Guangdong) were located in south central China. Shanxi, Liaoning, Taiwan, Hong Kong, and Guangxi are the provinces where our models were not able to reduce the error compared to the baseline. While ARGONet + GLEAM’s performance in these provinces was not superior to the baseline, its predictions were within a reasonable range, as seen in Figures 2 and 3. We were not able to perform any analysis on Tibet, one of the largest provinces in China, and Macau given their low count of detected COVID-19 cases.

### Analysis of the Importance of the Sources of Information Over Time

**Figure 8:**
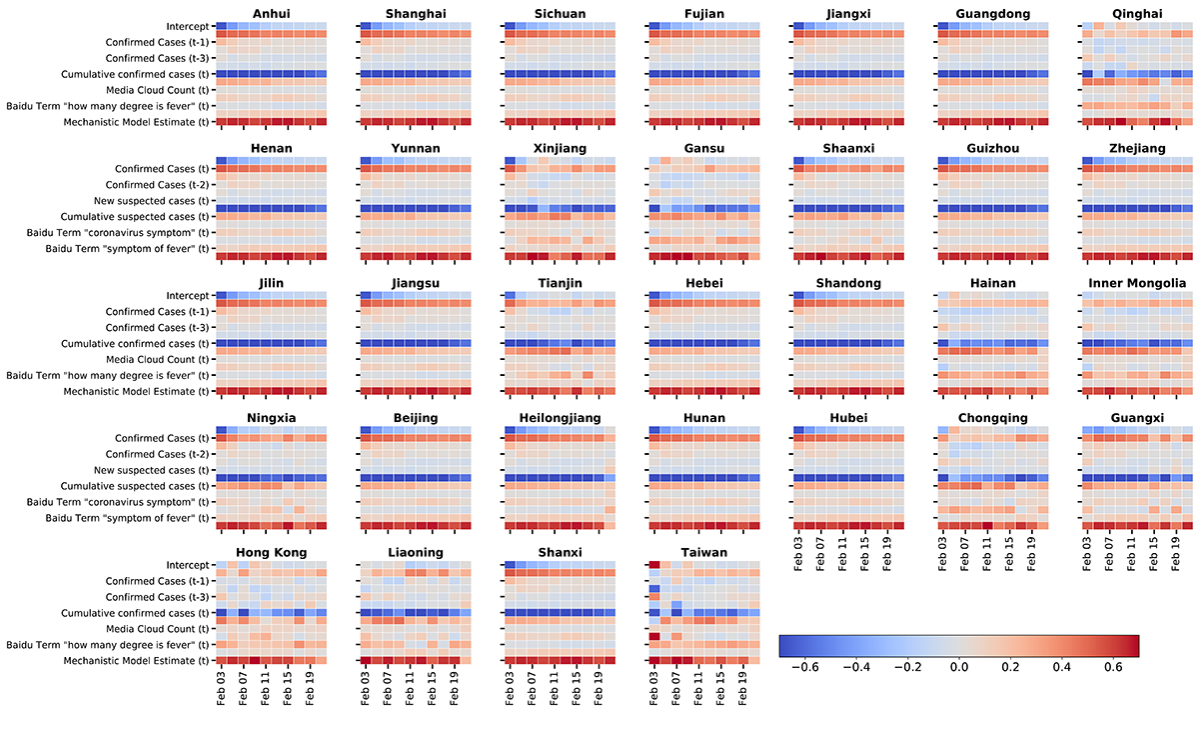
Graphical visualization of the relevance of data sources. Time evolution of the value (averaged over the 20 experiments) of the linear coefficients for the features used in our methodology, visualized per province. Every heatmap includes the same number of features (rows) and is organized in the same order.

To minimize the prediction errors in our estimates, the dynamic design of our methodology utilizes different sources of information as needed over time. This means that for each province (or group of provinces within a cluster), we can quantify the predictive power of different features used in our models as time evolves. Our analysis, visualized in Figure 8, shows that historical COVID-19 confirmed cases and suspected cases were consistently relevant sources of information over most of the study period. Internet-based search terms from Baidu were also frequently used. Daily news counts were used by our models in a selected number of provinces. However, for many of these provinces, the importance of media article counts decrease over time. Estimates from mechanistic models contributed to our model prediction, especially in early February 2020.

## Discussion

### Principal Findings

We presented a methodology capable of producing meaningful and reliable short-term (2 days ahead) forecasts of COVID-19 activity, at the province level in China, by combining information from reports from China CDC, internet search trends, news article trends, and information from mechanistic models. Our approach is capable of overcoming multiple challenges characteristic of emerging outbreaks caused by novel pathogens. These challenges include the lack of historical disease activity information to calibrate models, the low volume of case count data, and the inherent delay in gaining access to data. Methodologically speaking, our method maximizes the use of a limited number of observations as the outbreak unfolded by (a) choosing an appropriate aggregation time-window (2 days) to improve the signal-to-noise ratio, (b) leveraging synchronicities in the spatiotemporal trends in COVID-19 across provinces to produce cluster-specific models of prediction, and (c) using data augmentation methods to increase stability in the training of our models.

Previous methods, such as the ARGONet model [11,45], have been shown to make accurate real-time prediction at the state level in the United States for seasonal infectious diseases such as influenza. In addition, Chinazzi et al [16] showed that it was possible to estimate the evolution of an emerging outbreak using a mechanistic model. Nevertheless, as far as we know, reliable real-time methodologies to forecast new case counts for an emerging disease outbreak remained an unsolved problem. In this study, we showed that a dynamically trained machine learning model can accurately produce real-time estimates for COVID-19 outbreaks.

In terms of prediction error, our proposed methodology, ARGONet + GLEAM, was able to outperform the persistence model in 27 out of 32 provinces. While our method does not show prediction error improvements in Guangxi, Liaoning, Shanxi, Taiwan, and Hong Kong, our forecasts are still within range in all provinces except for Taiwan, where very few cases were reported during the time period of this study. It is important to note that Taiwan, Hong Kong, and Guangxi have different administrative (and likely health care) systems compared with the other provinces. This could explain the differences in COVID-19 trends in these regions and could help explain why our models do not seem to add value to the persistence model. Features studies should investigate if incorporating disease activity estimates from other mechanistic models, likely designed with different assumptions and mathematical formulations, could lead to further improvements.

We were unable to identify an accurate (daily) parametrization of changes in human mobility due to the widespread local lockdowns during the period of our study (February 3-21, 2020), and thus, we did not include this data source as a potential predictor. Future studies may incorporate (high temporal resolution) human mobility data as a modulator of transmission and predictor of disease activity. When looking at the entire time period of this study, however, we observed that the data-driven clustering of provinces used in our approach and based on COVID-19 activity appears to have similarities with the clustering one would obtain from using human mobility data made available by Baidu (Figure 5). This result aligns with the conclusion of other available studies that found that the time evolution of the COVID-19 outbreak in China was significantly influenced by changes in human mobility (consequence of public health interventions) [16,17,46], and associated with the percentage of people traveling from Wuhan in the early stages.

### Limitations

One limitation of our study is that during the test time period of our methods a consistent decrease in COVID-19 cases (due to strong public health interventions) was observed and thus our methods could not be tested for their ability to identify the epidemic peaks across provinces. The brevity of the COVID-19 epidemic outbreak in Chinese provinces was the limiting factor for this as the observations that corresponded to the growth phase of the outbreak were used for training purposes. Future model implementations in other locations where the growth phase has spanned longer time periods, like New York, United States, should investigate the ability of our models to properly identify peaks.

### Conclusions

Our findings suggest that it is possible to use very limited amounts of data from multiple data sources to conduct real-time forecasting in the early stage of an emerging outbreak. We believe that our method, ARGONet + GLEAM, could prove to be useful for public health officials to monitor (and perhaps prevent) the spread of the virus [8,11,25,47]. As the SARS-CoV-2 virus continues to spread around the world, extensions of our methods could be implemented to provide timely and reliable disease activity estimates to decision makers.

## Appendix

Multimedia Appendix 1 Mobility data correlation.

Multimedia Appendix 2 Supplementary tables.

## Abbreviations

AR: autoregressive
China CDC: Chinese Center for Disease Control and Prevention
COVID-19: coronavirus disease
GLEAM: global epidemic and mobility
I: infectious
L: latent
LASSO: least absolute shrinkage and selection operator
MIDAS: Models of Infectious Disease Agent Study
PHEIC: Public Health Emergency of International Concern
R: removed
RMSE: root mean square error
S: susceptible
SARS-CoV-2: severe acute respiratory syndrome coronavirus 2
WHO: World Health Organization
