# Correction: Real-Time Forecasting of the COVID-19 Outbreak in Chinese Provinces: Machine Learning Approach Using Novel Digital Data and Estimates From Mechanistic Models

**DOI:** 10.2196/23996

**Published:** 2020-09-22

**Authors:** Dianbo Liu, Leonardo Clemente, Canelle Poirier, Xiyu Ding, Matteo Chinazzi, Jessica Davis, Alessandro Vespignani, Mauricio Santillana

**Affiliations:** 1 Computational Health Informatics Program Boston Children’s Hospital Boston, MA United States; 2 Department of Pediatrics Harvard Medical School Boston, MA United States; 3 Tecnologico de Monterrey Monterrey Mexico; 4 Harvard TH Chan School of Public Health Boston, MA United States; 5 Laboratory for the Modeling of Biological and Socio-technical Systems Northeastern University Boston, MA United States; 6 ISI Foundation Turin Italy

In “Real-Time Forecasting of the COVID-19 Outbreak in Chinese Provinces: Machine Learning Approach Using Novel Digital Data and Estimates From Mechanistic Models” (J Med Internet Res 2020;22(8):e20285) the authors noted three errors.

The order of authors in the original article was listed as:

Canelle Poirier, Dianbo Liu, Leonardo Clemente, Xiyu Ding, Matteo Chinazzi, Jessica Davis, Alessandro Vespignani, Mauricio Santillana

The correct order of authors is:

Dianbo Liu, Leonardo Clemente, Canelle Poirier, Xiyu Ding, Matteo Chinazzi, Jessica Davis, Alessandro Vespignani, Mauricio Santillana

As well, degree information for authors Leonardo Clemente, Xiyu Ding, and Jessica Davis was listed incorrectly as "MD" in the original article. The correct degree for Leonardo Clemente and Xiyu Ding is "MSc"; the correct degree for Jessica Davis is "BSc".

The correction will appear in the online version of the paper on the JMIR Publications website on September 22, 2020, together with the publication of this correction notice. Because this was made after submission to PubMed, PubMed Central, and other full-text repositories, the corrected article has also been resubmitted to those repositories.

