# Supplementary material for: Real-Time Forecasting of the COVID-19 Outbreak in Chinese Provinces: Machine Learning Approach Using Novel Digital Data and Estimates From Mechanistic Models
Source: J Med Internet Res. 2020 Aug 17;22(8):e20285. doi: 10.2196/20285 (PMC7459435; doi:10.2196/20285)
Supplement: Multimedia Appendix 1 [file jmir_v22i8e20285_app1.docx]

# Multimedia Appendix 1

### Mobility data correlation

#### Human mobility data

As an additional source, we collected the daily human mobility (in terms of percentage) between the Chinese provinces via Baidu. Baidu mobility data was obtained from a public available archive (https://doi.org/10.18130/V3/YQLJ5W), which was originally scraped from Baidu website (qiangxi.baidu.com). The dataset contains daily inbound (i.e. percentage of people traveling to the city from all the cities in China) and outbound (i.e. percentage of people traveling from the city to all the cities in China) mobility data for all cities (except Hong Kong, Macau and Taiwan) in China on each day from January 1, 2020 to January 31, 2020. We decided to not include human mobility information as a source of information for our methodology given the absence of data during February.

However, we were particularly interested in the relationship between the amount of people traveling from Wuhan/Hubei to other provinces and the number of new confirmed cases in those provinces. Given the original data from Baidu is city-level, we first extracted all the outbound data from Wuhan (which is the capital of Hubei and the first city with confirmed COVID-19 cases). Then, we created a 31x31 matrix, where each row was a province in China, and each column was one day in January, each element was the percentage of people traveling out of Wuhan to the other provinces, among all people traveling from Wuhan on that day.

To understand the similarity of the human mobility from Wuhan among Chinese provinces we calculated the pairwise correlation matrix for the percentage of people traveling from Wuhan to each province by using the data from January 1, 2020 to January 31, 2020 (see Figure S1). If the pairwise correlation between the two provinces is high, it indicates that the trend in the percentage of people traveling from Wuhan to the two provinces are similar. Similarly, we calculated the pairwise correlation matrix for new confirmed cases using data from February 1, 2020 to February 21, 2020. Figure S1 shows both matrices, the pairwise correlation of human mobility and the pairwise correlation of COVID-19 case counts. Most of provinces highly correlated for case counts are also correlated for mobility. To further explore the potential impact of mobility on the new confirmed cases of each province, we calculated the Pearson correlation and cosinus similarity between the two correlation matrices. To do this, we first converted the two matrices into vectors and then calculated the correlation between both. We got Pearson correlation of 0.1473 and cosinus similarity of 0.4729.

### Clustering

Clustering is an unsupervised method of machine learning, allowing to classify each data point into a specific group. The aim of the clustering is to have the most similar data points in a same group and the most dissimilar data points in different groups. There are several algorithms of clustering including K-Means clustering, DBSCAN or Agglomerative Hierarchical Clustering [44]. In this study, we used complete linkage hierarchical clustering, which is an agglomerative hierarchical clustering method, creating clusters based on most dissimilar pairs [40]. To do this, at the first step of the algorithm, each data point represents a cluster. Then, for each following step, the two most similar clusters are combined. In our method, the number of clusters K was determined by choosing the K, maximizing Calinski-Harabasz index which is the ratio between intergroup and intragroup variances [41].

### Lasso Regression

A linear regression is a statistical method modelling the linear relationship between a dependent variable (variable to be explained) and independent variables (explanatory variables). The formulation of a linear regression model can be written as follows :

$$y_{t}=\sum_{i=1}^{n} \beta_{i}x_{it}+\epsilon_{t}$$

where Y the dependent variable and X={x_1_,x_2_,..x_n_} the independent variables. In the linear regression approach, parameters of the models are estimated with the least squares method consisting by minimizing the sum of the squared deviations between the real values of y_t_ and the values of y_t_ estimated by the model [45].

In this study, we used a Lasso regression, which is a penalized version of the linear regression. The parameters of the models are estimated with the least squares method but by adding an additional constraint corresponding to $\sum_{i=1}^{n} {|\beta}_{i}|<t$. This constraint, called L_1_ norm, tends to produce some coefficients that are exactly 0 and decreases the number of explanatory variables by selecting the most important inputs [46].

### Autoregressive model

An autoregressive model (AR) was built using only COVID-19 local (in contrast to ARGONet’s methodology) historical confirmed case counts from the past three two-day reports. Based on this formulation, our two-day forecast ($\hat{y_{t+2}}$) of COVID-19 confirmed cases is estimated as follows:

$$y_{T+\delta t}=\sum_{i=0}^{3} \alpha_{i}y_{T-i\delta t}+\epsilon_{T+\delta t}$$

where

• $y_{T+\delta t}$ is the estimate at date *T + δt*, where *δt = 2 days*

• *y_T_* is the number of cases at date *T*.

• $\epsilon_{T}$ ∼ 𝒩(0, σ² ), residuals

As for ARGONet+GLEAM, our version of AR was dynamically trained.
