# Supplementary material for: Real-Time Forecasting of the COVID-19 Outbreak in Chinese Provinces: Machine Learning Approach Using Novel Digital Data and Estimates From Mechanistic Models
Source: J Med Internet Res. 2020 Aug 17;22(8):e20285. doi: 10.2196/20285 (PMC7459435; doi:10.2196/20285)
Supplement: Multimedia Appendix 2 [file jmir_v22i8e20285_app2.docx]

# Multimedia Appendix 2

Table S1: Performance of the algorithm after different numbers of realizations of the whole process from clustering to out-of-sample prediction.

| Number of realizations | Number of provinces better than baseline by RMSE | Number of provinces better than baseline by correlation |
| --- | --- | --- |
| 1 | 25 | 25 |
| 2 | 26 | 26 |
| 5 | 29 | 27 |
| 10 | 29 | 28 |
| 15 | 28 | 28 |
| 20 | 28 | 28 |
| 25 | 28 | 28 |
| 30 | 28 | 28 |
| 35 | 28 | 28 |
| 40 | 28 | 28 |
| 45 | 28 | 28 |
| 50 | 28 | 28 |

Table S2: Detailed Results of the implemented models in each Chinese Province.

| **Location** | **Metric** | **Baseline** | **AR** | **ARGONet** | **ARGONet + Mechanistic** |
| --- | --- | --- | --- | --- | --- |
| Anhui | RMSE | 21.10 | 21.84 | 11.27 | 9.86 |
|  | Pearson | 0.90 | 0.92 | 0.97 | 0.98 |
| Shanghai | RMSE | 8.20 | 9.52 | 5.42 | 3.84 |
|  | Pearson | 0.82 | 0.83 | 0.79 | 0.89 |
| Sichuan | RMSE | 7.20 | 8.39 | 5.49 | 4.34 |
|  | Pearson | 0.86 | 0.88 | 0.86 | 0.90 |
| Fujian | RMSE | 4.70 | 6.54 | 3.65 | 2.86 |
|  | Pearson | 0.92 | 0.93 | 0.94 | 0.95 |
| Jiangxi | RMSE | 19.40 | 19.21 | 12.60 | 11.82 |
|  | Pearson | 0.89 | 0.94 | 0.95 | 0.96 |
| Guangdong | RMSE | 23.70 | 25.80 | 19.07 | 14.91 |
|  | Pearson | 0.96 | 0.95 | 0.93 | 0.96 |
| Qinghai | RMSE | 1.10 | 1.59 | 0.65 | 0.69 |
|  | Pearson | 0.45 | 0.35 | 0.91 | 0.87 |
| Henan | RMSE | 21.80 | 25.02 | 18.11 | 14.14 |
|  | Pearson | 0.93 | 0.94 | 0.96 | 0.98 |
| Yunnan | RMSE | 3.90 | 5.12 | 3.26 | 2.69 |
|  | Pearson | 0.66 | 0.73 | 0.70 | 0.72 |
| Xinjiang | RMSE | 1.80 | 1.96 | 1.38 | 1.33 |
|  | Pearson | 0.67 | 0.70 | 0.85 | 0.87 |
| Gansu | RMSE | 4.70 | 5.20 | 3.63 | 3.48 |
|  | Pearson | 0.37 | 0.37 | 0.67 | 0.68 |
| Shaanxi | RMSE | 4.40 | 5.62 | 4.06 | 3.32 |
|  | Pearson | 0.90 | 0.92 | 0.85 | 0.91 |
| Guizhou | RMSE | 4.10 | 3.96 | 3.02 | 3.17 |
|  | Pearson | 0.83 | 0.84 | 0.90 | 0.88 |
| Zhejiang | RMSE | 23.60 | 26.36 | 23.62 | 18.46 |
|  | Pearson | 0.89 | 0.96 | 0.81 | 0.87 |
| Jilin | RMSE | 3.70 | 3.28 | 3.40 | 2.99 |
|  | Pearson | 0.70 | 0.73 | 0.73 | 0.81 |
| Jiangsu | RMSE | 7.60 | 8.36 | 5.70 | 6.16 |
|  | Pearson | 0.95 | 0.96 | 0.97 | 0.98 |

Table S3: Detailed Results of the implemented models in each Chinese Province.

|  |  | **Baseline** | **AR** | **ARGONet** | **ARGONet + Mechanistic** |
| --- | --- | --- | --- | --- | --- |
| **Tianjin** | RMSE | 4.40 | 4.17 | 3.58 | 3.60 |
|  | Pearson | 0.43 | 0.42 | 0.46 | 0.53 |
| **Hebei** | RMSE | 9.40 | 8.91 | 8.60 | 7.79 |
|  | Pearson | 0.62 | 0.68 | 0.78 | 0.81 |
| **Shandong** | RMSE | 30.10 | 33.59 | 28.31 | 25.89 |
|  | Pearson | -0.13 | -0.06 | -0.12 | -0.08 |
| **Hainan** | RMSE | 4.60 | 4.98 | 4.59 | 4.00 |
|  | Pearson | 0.78 | 0.81 | 0.76 | 0.84 |
| **Inner Mongolia** | RMSE | 3.20 | 10.66 | 2.89 | 2.80 |
|  | Pearson | 0.40 | -0.56 | 0.13 | 0.24 |
| **Ningxia** | RMSE | 2.30 | 2.33 | 2.34 | 2.03 |
|  | Pearson | 0.57 | 0.61 | 0.65 | 0.73 |
| **Beijing** | RMSE | 5.90 | 6.89 | 6.36 | 5.24 |
|  | Pearson | 0.93 | 0.94 | 0.86 | 0.91 |
| **Heilongjiang** | RMSE | 10.50 | 9.20 | 9.96 | 9.49 |
|  | Pearson | 0.84 | 0.86 | 0.89 | 0.94 |
| **Hunan** | RMSE | 12.80 | 18.12 | 10.52 | 11.59 |
|  | Pearson | 0.97 | 0.97 | 0.97 | 0.97 |
| **Hubei** | RMSE | 3987.70 | 3542.12 | 3198.93 | 3808.56 |
|  | Pearson | -0.07 | -0.01 | 0.16 | 0.22 |
| **Chongqing** | RMSE | 10.00 | 14.16 | 10.72 | 9.65 |
|  | Pearson | 0.86 | 0.80 | 0.62 | 0.74 |
| **Guangxi** | RMSE | 4.60 | 5.20 | 5.06 | 5.10 |
|  | Pearson | 0.85 | 0.88 | 0.83 | 0.85 |
| **Hong Kong** | RMSE | 2.90 | 2.66 | 2.89 | 3.23 |
|  | Pearson | 0.37 | 0.35 | 0.48 | 0.49 |
| **Liaoning** | RMSE | 3.30 | 3.54 | 4.99 | 4.35 |
|  | Pearson | 0.79 | 0.88 | 0.22 | 0.68 |
| **Shanxi** | RMSE | 2.00 | 2.71 | 3.39 | 2.69 |
|  | Pearson | 0.89 | 0.91 | 0.88 | 0.93 |
| **Taiwan** | RMSE | 1.30 | 1.13 | 1.50 | 2.51 |
|  | Pearson | 0.06 | -0.14 | 0.18 | -0.31 |
